# Impact of environmental factors on *Biomphalaria pfeifferi* vector capacity leading to human infection by *Schistosoma mansoni* in two regions of western Côte d'Ivoire

**DOI:** 10.1186/s13071-024-06163-2

**Published:** 2024-04-05

**Authors:** Edwige A. Sokouri, Bernardin Ahouty Ahouty, Martial N’Djetchi, Innocent A. Abé, Ble Gbacla Flora Dominique Yao, Thomas Konan Konan, Annette MacLeod, Harry Noyes, Oscar Nyangiri, Enock Matovu, Mathurin Koffi, the TrypanoGEN+ Research Group of the H3Africa Consortium

**Affiliations:** 1 Laboratoire de Biodiversité et Gestion des Ecosystèmes Tropicaux, Unité de Recherche en Génétique et Epidémiologie Moléculaire, UFR Environnement, Université Jean Lorougnon Guédé, Daloa, Côte d’Ivoire; 2 College of Medical, Veterinary and Life Sciences, Institute of Biodiversity Animal Health and Comparative Medicine, University of Glasgow, Liverpool, UK; 3 Centre for Genomic Research, University of Liverpool, Liverpool, UK; 4 College of Veterinary Medicine, Animal Resources and Biosecurity, Makerere University, Kampala, Uganda

**Keywords:** Environmental factors, *Biomphalaria pfeifferi*, *Schistosoma mansoni*, Thermophilic coliforms, Western Côte d’Ivoire

## Abstract

**Background:**

Intestinal schistosomiasis remains a worrying health problem, particularly in western Côte d'Ivoire, despite control efforts. It is therefore necessary to understand all the factors involved in the development of the disease, including biotic and abiotic factors. The aim of this study was to examine the factors that could support the maintenance of the intermediate host and its vectorial capacity in western Côte d'Ivoire.

**Methods:**

Data on river physicochemical, microbiological, and climatic parameters, the presence or absence of snails with *Schistosoma mansoni*, and human infections were collected between January 2020 and February 2021. Spearman rank correlation tests, Mann–Whitney, analysis of variance (ANOVA), and an appropriate model selection procedure were used to analyze the data.

**Results:**

The overall prevalence of infected snails was 56.05%, with infection reaching 100% in some collection sites and localities. Of 26 sites examined, 25 contained thermophilic coliforms and 22 contained *Escherichia coli*. *Biomphalaria pfeifferi* was observed in environments with lower land surface temperature (LST) and higher relative air humidity (RAH), and *B. pfeifferi* infection predominated in more acidic environments. Thermal coliforms and *E. coli* preferred higher pH levels. Lower maximum LST (LST_Max) and higher RAH and minimum LST (LST_Min) were favorable to *E. coli*, and lower LST_Max favored coliforms. The presence of *B. pfeifferi* was positively influenced by water temperature (*T* °C), LST_Min, RAH, and precipitation (Pp) (*P* < 0.05) and negatively influenced by pH, total dissolved solids (TDS), electrical conductivity (EC), LST_Max, and mean land surface temperature (LST). The parameters pH, TDS, EC, LST_Min, LST, and Pp had a positive impact on snail infection, while LST_Max had a negative impact on infection. Only pH had a positive effect on coliform and *E. coli* abundance. Of the 701 people examined for human schistosomiasis, 73.13% were positive for the point-of-care circulating cathodic antigen (POC-CCA) test and 12.01% for the Kato–Katz (KK) test. A positive correlation was established between human infections and the abundance of *Biomphalaria* (*r*^2^ = 0.879, *P* = 0.04959).

**Conclusions:**

The results obtained reflect the environmental conditions that are conducive to the maintenance of *S. mansoni* infection in this part of the country. To combat this infection as effectively as possible, it will be necessary not only to redouble efforts but also to prioritize control according to the level of endemicity at the village level.

**Graphical Abstract:**

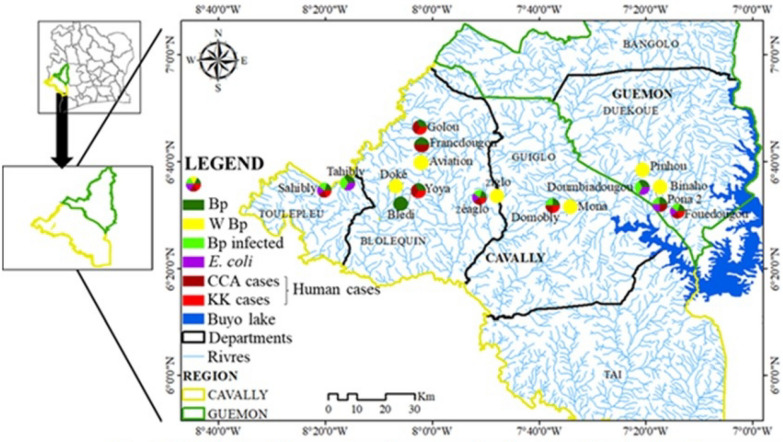

## Background

Schistosomiasis is one of the most prevalent neglected tropical diseases (NTDs) of water-borne origin and represents the second most important parasitic disease after malaria [1, 2]. Schistosomiasis is endemic in particular in areas where populations are dependent on non-sanitized and contaminated surface water [3]. Humans become infected when they are exposed to fecally contaminated water that serves as a habitat for the parasite’s intermediate host, mainly freshwater snails of the genera *Biomphalaria*, *Bulinus*, and *Oncomelania* [4, 5].

In Côte d'Ivoire, intestinal schistosomiasis is one of the NTDs commonly observed in the western part of the country [6]. It is caused by *Schistosoma mansoni*, whose known intermediate host is *Biomphalaria pfeifferi* [7, 8]. As in many bilharzia-affected countries, control of the disease relies on mass administration of the drug praziquantel [2]. However, to date, this strategy has not been successful in mitigating the infection burden, which is expanding in Côte d'Ivoire [9] despite the efforts of the schistosomiasis national control program. Indeed, the persistence of the disease cycle and its spread in the population are both the result of unhygienic conditions, lack and inadequacy of sanitation, cultural habits, and domestic and professional work, exacerbated by the presence of the snail *B. pfeifferi* in western Côte d'Ivoire [4, 10]. Interruption of schistosomiasis transmission therefore requires the complete integration of the different factors involved in the disease cycle [11]

This process requires an understanding of all the factors involved, including biotic factors (*S. mansoni*, *B. pfeifferi*, human) and abiotic factors (climate, vegetation, physicochemical parameters of fresh water, etc.). This understanding will allow us to prioritize the risk of transmission and consequently to adapt the strategy to fight and control this infection in endemic regions.

The overall objective of this study is to contribute to the elimination of schistosomiasis in Côte d'Ivoire. More specifically, we will examine the factors that could support the maintenance of the intermediate host and its vectorial capacity.

## Methods

### Study area

**Fig. 1:**
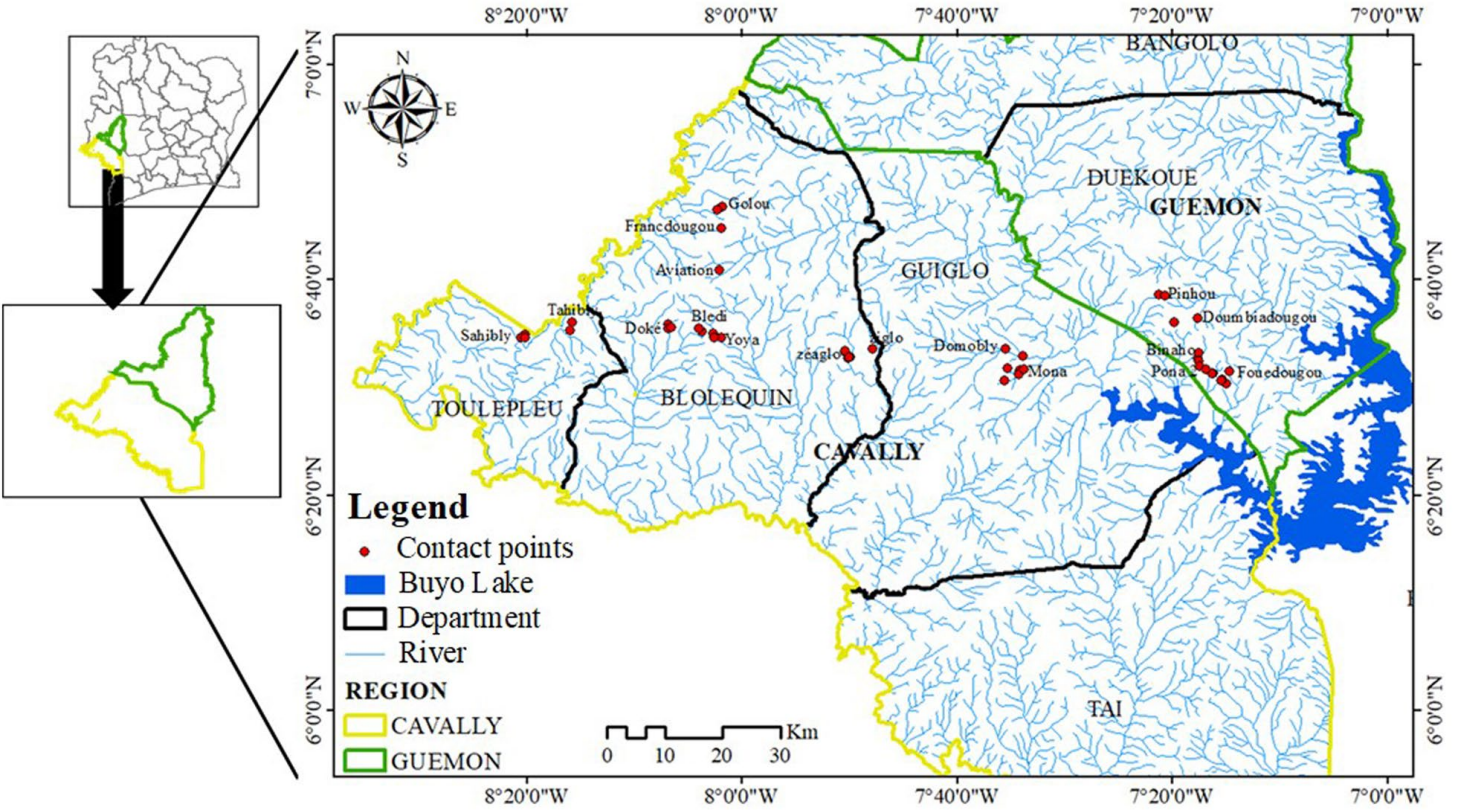
Map of the location of sampling sites in the study area

The study was carried out between January 2020 and February 2021 in 17 villages in four departments belonging to two regions in western Côte d'Ivoire that are endemic for intestinal bilharziasis (Fig. 1). Populations from these villages live mainly by agriculture, artisanal fishing, and gold panning. Some of the main environmental problems facing the population are open waste, lack of access to drinking water, inadequate latrines, and poor sanitation [10].

### Collection of abiotic parameters: physicochemical and climatic parameters

Data on the physicochemical parameters of water quality were collected at the sampling sites (Fig. 1). Using a multiparameter probe (Hanna Instruments, Møllevången, Sweden), data on the water temperature (*T* °C), hydrogen potential (pH), total dissolved solids (TDS), and electrical conductivity of the water (EC) were collected at each contact point (CP). Climatic data, including the minimum land surface temperature (LST_Min), maximum land surface temperature (LST_Max), mean land surface temperature (LST), relative air humidity (RAH), and precipitation (Pp), were obtained from the National Aeronautics and Space Administration (NASA) website (https://power.larc.nasa.gov/data-access-viewer/) (consulted 29/07/2022).

### Malacological survey

Snails were collected with a dip net and tongs. Collection by dip net was performed by dipping the strainer in water and gently shaking the submerged plants that were difficult to access. Alternatively, the snails were collected by detaching them from the submerged plants and supports with a pair of tongs. The search for snail vectors at each CP was performed by two collectors over a period of 15 min, which could be repeated once in the case of low snail density [12]. Snails were identified by observation [13], and only schistosomiasis vectors for each CP were carefully stored in plastic boxes and transferred to the laboratory for infection testing.

### Excretion of cercariae

Snails collected in the field and brought back to the laboratory were individually placed in glass tubes containing 10 ml of borehole water or mineral water [14]. They were then exposed to artificial light for 4 h [4, 15, 16]. This lighting device generates a temperature between 28 and 30 °C capable of triggering the excretion of *S. mansoni* cercariae [4]. The pillboxes were taken to a binocular stereomicroscope for observation of the cercariae [12, 17], and identification was performed as described previously [13, 17]. This enabled the confirmation of schistosomiasis infection and identification of schistosomiasis transmission foci. Between the excretion stimulation experiments, negative snails were kept in aquaria at room temperature, where they were fed lettuce and fish weed to allow parasites to develop. Positive snails were set aside.

### Fecal contamination

Water samples of about 100 ml were collected in Stomacher bags to determine the presence of fecal contamination. Violet red bile lactose (VRBL) agar culture medium [18–20] was used to test for the presence of thermophilic coliforms, and RAPID’E.coli medium (Bio-Rad, France) [21, 22] was used to confirm the presence of *Escherichia coli*. Quantification was performed per colony-forming unit (CFU).

### Parasitological and serological survey of intestinal schistosomiasis

Populations from villages with rivers containing snails of the genus *Biomphalaria* were surveyed for *S. mansoni* infection. Each individual who agreed to participate in the study was given two sterile polypropylene sampling bottles for sample collection: one for the morning fresh stool sample (about 5 g) and the second for the morning fresh urine (about 40 ml). Parasitological diagnosis was carried out using the Kato–Katz (KK) test [23, 24]. In practice, two thick KK smears were prepared from the stool samples of each participant. The smears were then examined microscopically by two technicians for the detection and quantification of *S. mansoni* eggs [23, 25]. Eggs were counted and expressed as eggs per gram (EPG). Infections were classified according to the World Health Organization (WHO) recommendations [25].

The serological test consisted of the point-of-care circulating cathodic antigen (POC-CCA) test (ICT Diagnostics, Cape Town, South Africa). This test detects and quantifies the circulating cathodic antigens (CCA) of *S. mansoni*. First, two drops of fresh urine are inoculated into the well of the cassette, and after 20 min of complete absorption and incubation, a visual (semi-quantitative) result is obtained. This result is based on the absence/presence and intensity of coloration of the test line. It is scored as follows: negative (absence of test line staining), trace, +1, +2, +3 (depending on the intensity of test line staining) [26, 27]. To assess the intensity of infection, the incubated cassette is systematically introduced into the ESEQuant LR3 reader (Qiagen, Germany) designed for quantification of target analytes on lateral flow test strips, including CCA strips. The ESEQuant LR3 reader is pre-calibrated using samples of different concentrations of CCA supplied by the University of Leiden, Netherlands, implemented in Lateral Flow Studio (LF Studio) software. Finally, a correlation is established between the amount of antigen (CCA) and the intensity of the staining on the strip, which is expressed in millivolts for each sample. The millivolt value is therefore considered to be a relative measure of the amount of CCA present in the urine sample [26, 27].

### Statistical analysis

Data mining and regression modeling were performed using R software (version 4.2.2).

Each climatic and physicochemical variable was analyzed using a descriptive statistics test. Spearman’s rank correlation test was performed to evaluate the relationships between variables [28, 29]. In addition, the Mann–Whitney *U*-test was used to test the heterogeneity of climatic and physicochemical characteristics for study sites with and without the biotic factors (total *B. pfeifferi* collected, infected *B. pfeifferi*, thermophilic coliforms, and *E. coli*). A Shapiro–Wilk normality test was performed on the numerical data (shellfish abundance, CCA amount, *S. mansoni* egg number) to determine the type of distribution. Parametric (Student’s *t*-test) and non-parametric (Mann–Whitney and Kruskal–Wallis *U*-test) analysis of variance (ANOVA) tests were used to compare the different groups. All statistical tests were performed at the 0.05 significance level.

A stepwise top-down selection procedure was followed to build the model from the full model involving climatic, physicochemical, and biotic variables. The model with the lowest Akaike information criterion (AIC) value was selected as the optimal model [30]. The goodness of fit of the models was assessed using the relationship between the residuals and the predictor variables, and the normality of the residuals was tested using a quantile–quantile (QQ)-plot (probability plot) [31, 32]. The selected models were considered reliable only if no relationship between the residuals and the predictor variables was visually observed and the residuals were normally distributed.

### Ethical considerations and survey form

This study was approved by the National Ethics Committee on Life Sciences and Health (CNESVS) under the approval number N/Ref: 040-19/MSHP/CNESVS-kp. The study also received authorization approval for publication of data from this research (N/Ref: 187-23/MSHPCMU/CNESVS-km). In addition, the study was authorized by the regional, departmental, and village health and education authorities. Informed consent and assent forms were signed by the participants and their parents/guardians. Beforehand, information about the study’s objectives was provided to participants in French or in their own language. In addition, a survey form was completed by each participant to collect sociological data.

## Results

### Malacological survey

**Fig. 2:**
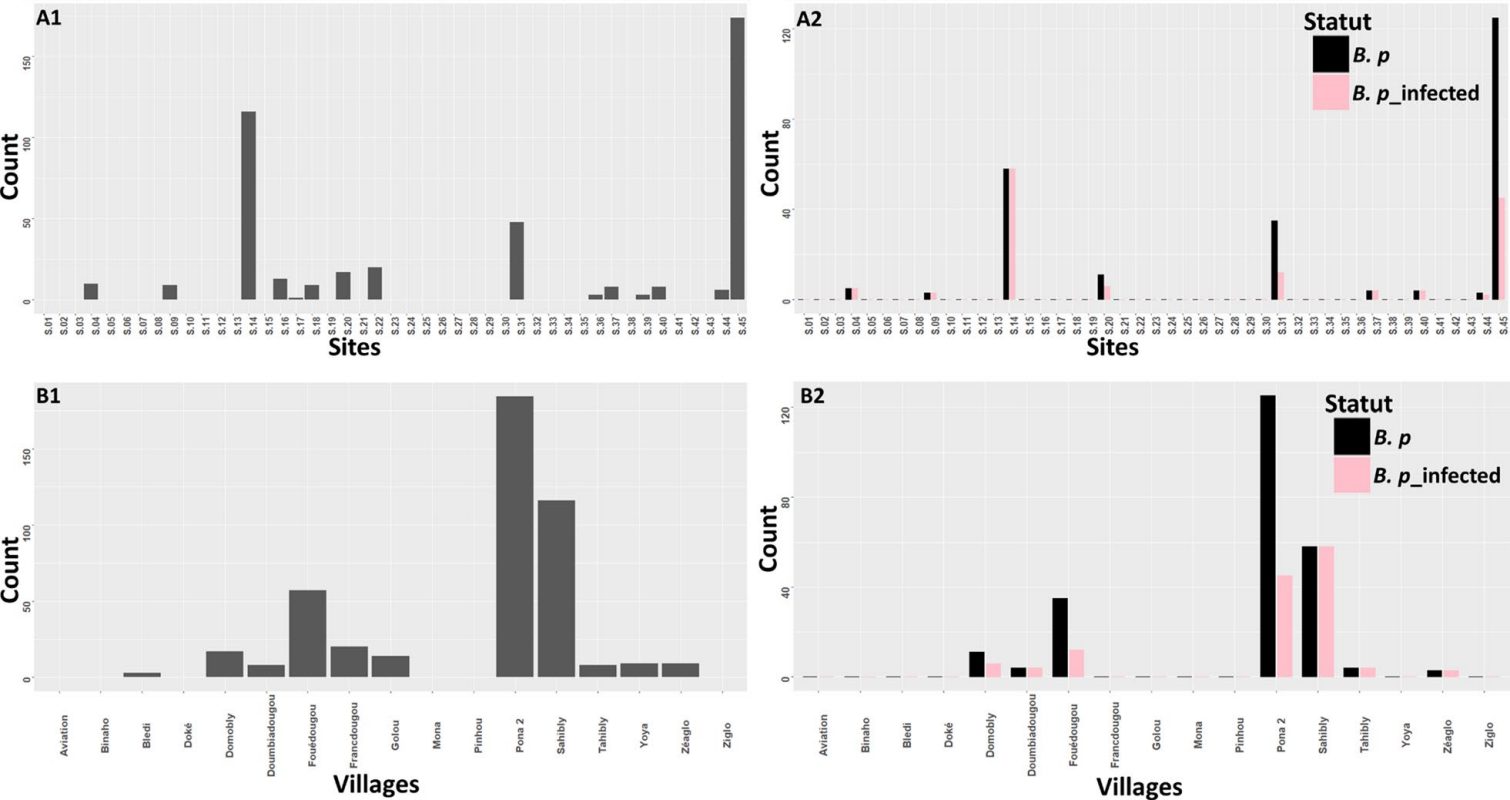
Distribution of prevalence of snails infected with *Schistosoma mansoni*. **A1** Number of collected snails by water–human contact points; **A2** prevalence of tested snails infected with *S. mansoni* by water–human contact points; **B1** number of collected snails by village; **B2** prevalence of tested snails infected with *S. mansoni* by village. B.p: total number of *Biomphalaria pfeifferi* tested; B. p_inf: number of *B. pfeifferi* infected with *S. mansoni*. AVI: aviation, BLE: Bledi, BIN: Binaho, DOK: Doke, DOM: Domobly, DOU: Doumbiadougou, FOU: Fouédougou, FRA: Francdougou, GOL: Golou, MON: Mona, PIN: Pinhou, PON: Pona, SAH: Sahibly, TAH: Tahibly, YOY: Yoya, ZEA: Zéaglo, ZIG: Ziglo

Among the 17 villages visited (Fig. 1), 45 CPs in 35 streams were surveyed during this study (Fig. 1). Of the 45 CPs, 15 contained snails of the species *B. pfeifferi*, and a total of 306 specimens of this species were collected. Apart from *B. pfeifferi*, other intermediate hosts of schistosomes were collected during the different surveys. These were *Bilunus globosus* (*n* = 51) and *Bilunus forskalii* (*n* = 22), but in small quantities. Of 248 *B. pfeifferi* examined by the cercarial emission test, 139 were infected with *S. mansoni*, yielding an overall prevalence of 56.05%. The prevalence of *S. mansoni* infection in snails at the CPs and in the localities ranged from 0 to 100% (Fig. 2). Limnic environments with the presence of *B. pfeifferi* in the Tahibly, Zéaglo, and Sahibly localities showed 100% infection.

### Fecal contamination

Microbial water quality analysis was performed at 26 CPs from 24 rivers. Of these 26 CPs, nine contained *Biomphalaria* and 17 had no *Biomphalaria*. Of the nine CPs containing *Biomphalaria*, analyses showed that only one CP was not contaminated with *E. coli* and thermophilic coliforms. For CPs without *Biomphalaria*, analyses showed that one CP was contaminated with thermophilic coliforms, while three other CPs were contaminated with *E. coli*. The prevalence of *E. coli* and thermophilic coliforms across all CPs was 84.61% and 92.31%, respectively. Bacteria counts ranged from 0 to 15 CFU for *E. coli* and 0 to 165 CFU for thermophilic coliforms.

### Spatial distribution of biotic factors in relation to intestinal schistosomiasis

**Fig. 3:**
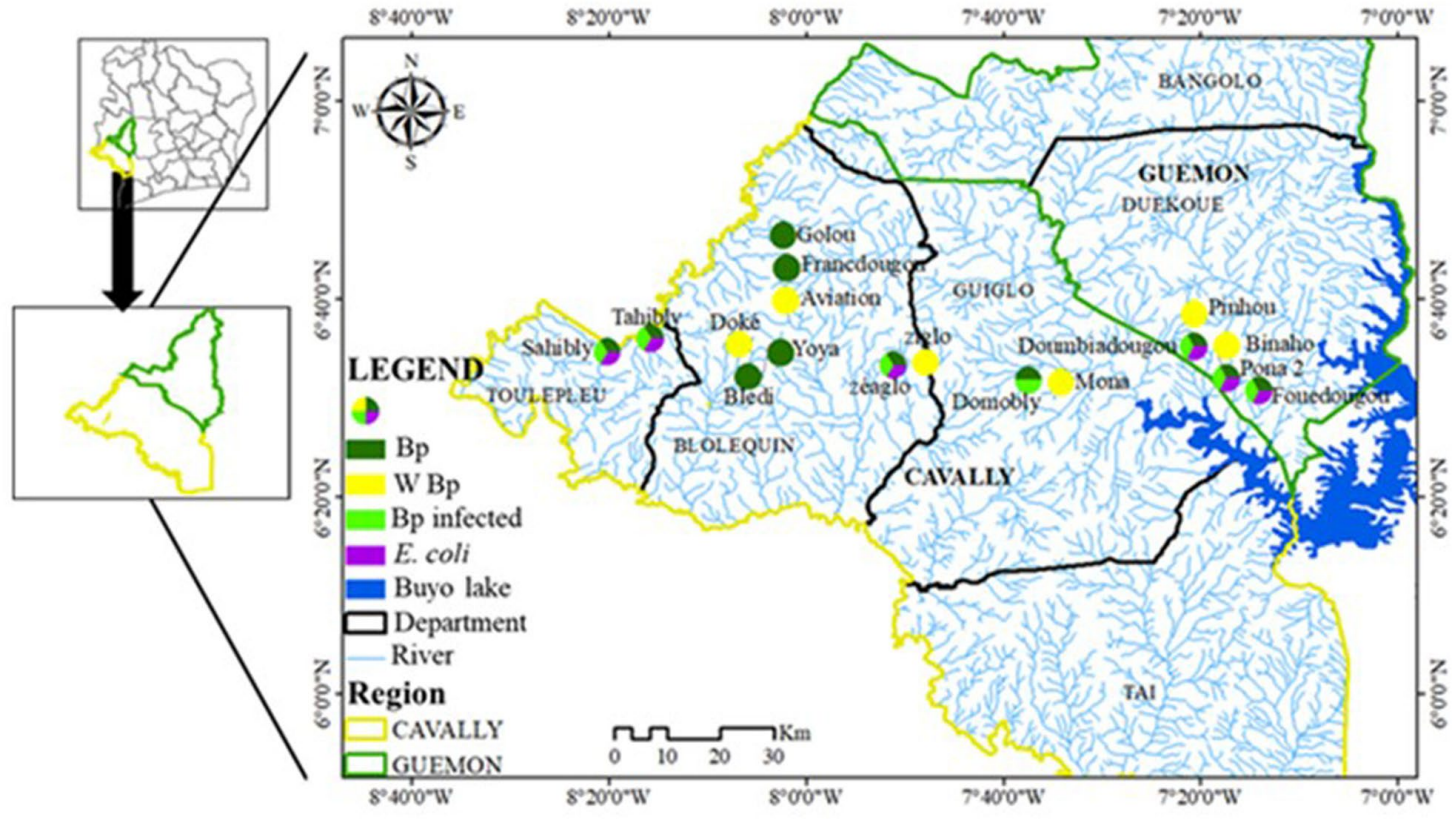
Distribution of environmental biotic parameters. Bp: *Biomphalaria pfeifferi*; W Bp: without *B pfeifferi*; Bp infected: *B. pfeifferi* infected with *S. mansoni*

Fifteen potential points of intestinal schistosomiasis transmission (presence of *B. pfeifferi*) were detected, and these belonged to 13 rivers affiliated to 11 villages (Figs. 2A, 3). The rate of potential transmission points per department was 50%, 60%, 62.5%, and 100% for the departments of Guiglo, Duékoué, Bloléquin, and Toulepleu, respectively. Snails from only 7 out of 11 villages were subjected to the cercarial emission test, and all of these villages had infected snails. These seven villages were spread across all the departments visited. This represents 100% presence of infected snails at the departmental level. Contamination of fecal origin was found in all the localities analyzed (Fig. 3), although contamination was not detected in certain rivers and CPs. The distribution of the risk of infection is therefore high in localities where *B. pfeifferi* and *E. coli* are present.

### Physicochemical characteristics of limnetic and climatic environments

With regard to descriptive parameters, the *T* °C recorded at the different CPs was between 21.70 and 32.10 °C. The pH was very acidic at some CPs and varied between 1.51 and 6.90. The climatic variables LST and Pp varied between 24.16 and 24.81 °C and between 00 and 16.97 mm, respectively. The RAH was above average (72–91.25%) (Table 1).

**Table 1:** Distribution of physicochemical and climatic characteristics of the surveyed sites

Parameters	Min	25%	50%	75%	Max	CV
*T* °C	21.70	24.50	26.00	27.50	32.10	0.087
pH	1.51	3.10	4.00	4.50	6.90	0.3258
TDS (ppm)	8.0	19.0	23.0	26.0	49.0	0.3318
CE (µScm^−1^)	16.0	38.0	47.0	51.5	98.0	0.3289
LST_Min (°C)	18.09	21.15	21.28	21.86	21.98	0.06299
LST_Max (°C)	27.95	28.02	28.58	28.67	30.87	0.03945
LST (°C)	24.16	24.34	24.50	24.55	24.81	0.007522
RAH (%)	72.38	86.94	90.00	91.00	91.25	0.08426
Pp (mm)	0.00	4.43	7.69	12.96	16.97	0.7435

Min: minimum; Max: maximum; CV: coefficient of variation; *T* °C: water temperature; pH: hydrogen potential; TDS: total dissolved solids; EC: electrical conductivity; LST_Min: minimum land surface temperature; LST_Max: maximum land surface temperature; LST: mean land surface temperature; RAH: relative air humidity; Pp: precipitation

### Influence of abiotic parameters on the presence or absence of all *B. pfeifferi*, infected *B. pfeifferi*, thermophilic coliforms, and *E. coli* at contact points

Analyses indicate that LST (*P* = 0.02) and RAH (*P* = 0.02) have a significant effect on the presence or absence of *B. pfeifferi* (Table 2). In addition, variation in pH acidity had a significant effect (*P* = 0.006) on the presence of infected *B. pfeifferi* at the CPs studied. The pH also had a significant influence on the presence or absence of thermophilic coliforms (*P* = 0.0382) and *E. coli* (*P* = 0.0031) at the CPs. LST_Min values were significantly different in sites with and without coliforms and *E. coli*. LST_Max and RAH values were significantly different in sites with and without *E. coli* (Table 2).

**Table 2:** Presence or absence of *Biomphalaria pfeifferi* (infected or not), thermophilic coliforms, and *E. coli* at the contact points studied as a function of abiotic parameters

Factors	Total *B. pfeifferi*			Total infected *B. pfeifferi*		
	Contact point (CP, *n* = 45)			Contact point (CP, *n* = 42)		
	Absence of *B. pfeifferi* (CP, *n* = 30)	Presence of *B. pfeifferi* (CP, *n* = 15)	*P*	Absence of infected *B. pfeifferi* (CP, *n* = 33)	Presence of infected *B. pfeifferi* (CP, *n* = 9)	*P*
*T* °C	25.7 (24.5, 27.6)	26 (24.40, 26.65)	0.89^a^	25.9 (24.5, 27.52)	26.5 (26, 28.5)	0.13^b^
pH	4 (3.48, 4.50)	4.06 (3.05, 5.65)	0.83^a^	4.03 (3.50, 4.50)	3.10 (2.70, 3.60)	0.00*^a^
TDS	24 (19.25, 26)	22 (19, 24)	0.42^b^	24 (19.75, 26)	22 (21, 24)	0.72^b^
EC	48 (39.25, 51.87)	43.7 (37.9, 48.5)	0.41^b^	48 (39.75, 51.62)	45 (43, 49)	0.75^b^
LST_Min	21.21 (19.15, 21.86)	21.42 (21.21, 21.86)	0.30^b^	21.28 (19.15, 21.86)	21.42 (21.15, 21.86)	0.37^b^
LST_Max	28.62 (28.02, 30.73)	28.02 (27.95, 28.62)	0.08^b^	28.58 (28.02, 30.73)	28.02 (27.96, 28.68)	0.15^b^
LST	24.50 (24.42, 24.55)	24.34 (24.25, 24.52)	0.02*^b^	24.34 (24.34, 24.54)	24.4 (24.3, 24.6)	0.20^b^
RAH	88.47 (74.38, 90.19)	91 (87.47, 91.12)	0.02*^b^	90 (74.38, 90.19)	91 (86.94, 91.25)	0.13^b^
Pp	8.64 (0.01, 12.96)	7.69 (6.06, 12.85)	0.8^b^	12.85 (0.01, 12.96)	7.69 (4.43, 12.87)	0.97^b^

Factors	Total thermophilic coliforms (TC)			Total *E. coli*		
	Contact point (CP, *n* = 26)			Contact point (CP, *n* = 26)		
	Absence of TC (CP, *n* = 2)	Presence of TC (CP, *n* = 24)	*P*	Absence of *E. coli* (CP, *n* = 4)	Presence of *E. coli* (CP, *n* = 22)	*P*
*T* °C	27.75 (27.02, 28.47)	26.55 (25.77, 29)	0.81^b^	28.7 (27.95, 28.97)	26.5 (25.72, 29.1)	0.39^b^
pH	2.07 (2.02, 2.12)	3.88 (3.38, 4.07)	0.04*^b^	2.07 (1.96, 2.32)	3.95 (3.49, 4.08)	0.00*^b^
TDS	22 (21, 23)	21.5 (18, 24)	0.66^b^	21.5 (19, 23.25)	21.25 (18.25, 24)	0.97^b^
EC	44.5 (42.8, 46.25)	43 (37.8, 48)	0.63^b^	44 (40.25, 47.25)	43 (37.25, 48)	0.80^b^
LST_Min	19.15 (19.15, 19.15)	21.35 (21.15, 21.86)	0.07^b^	19.15 (18.88, 19.68)	21.42 (21.15, 21.86)	0.02*^b^
LST_Max	30.9 (30.9, 30.9)	28.3 (28, 28.7)	0.04*^b^	30.8 (30.19, 30.87)	28.02 (27.97, 28.67)	0.04*^b^
LST	24.6 (24.6, 24.6)	24.5 (24.3, 24.6)	0.22^b^	24.53 (24.49, 24.55)	24.46 (24.34, 24.55)	0.35^b^
RAH	74.4 (74.4, 74.4)	90.1 (86.9, 91.1)	0.07^b^	74.38 (73.88, 78.33)	90.09 (86.94, 91.18)	0.04*^b^
Pp	0.01 (0.01, 0.01)	12.85 (4.43, 12.96)	0.07^b^	0.01 (0.007, 3.24)	12.85 (4.43, 12.96)	0.08^b^

The physicochemical and climatic parameters are presented in median form (first and third quantiles)

*T* °C: water temperature; pH: hydrogen potential; TDS: total dissolved solids; EC: electrical conductivity; LST_Min: minimum land surface temperature; LST_Max: maximum land surface temperature; LST: mean land surface temperature; RAH: relative air humidity; Pp: precipitation

*Indicates significance at *P* < 0.05

^a^Student’s *t* test

^b^Wilcoxon test

### Effects of abiotic characteristics on biotic factors

The results of the negative binomial generalized linear mixed-effects model (NB-GLMM) on abundance and presence/absence data are recorded in Table 3. Abiotic variables had no effect on the abundance of total *B. pfeifferi* and infected *B. pfeifferi*. However, the variables *T* °C, LST_Min, RAH, and Pp positively influenced (*P* < 0.05) the presence of *B. pfeifferi*, while pH, TDS, EC, LST_Max, and LST had a negatively influence. With regard to the presence of infected *B. pfeifferi*, the effects of pH, TDS, EC, LST_Min, LST, and Pp were positive, and that of LST_Max was negative. Only pH had a significant and positive effect on the abundance of both thermophilic coliforms and *E. coli*.

**Table 3:** Estimated effects of abiotic characteristics on biotic factors

Factors	Total *Biomphalaria pfeifferi*							
	Abundance				Presence			
	Estimate	Std. error	*z* value	*P*	Estimate	Std. error	*z* value	*P*
*T* °C	0.023	0.368	−0.064	0.949	0.307	0.063	4.871	< 0.001*
pH	−0.243	0.331	−0.735	0.462	−0.360	0.039	−9.104	< 0.001*
TDS	−0.038	0.109	−0.350	0.726	−0.175	0.015	−11.322	< 0.001*
EC	−0.017	0.055	−0.313	0.754	−0.086	0.007	−11.337	< 0.001*
LST_Min	0.360	0.426	0.845	0.398	0.659	0.056	11.80	< 0.001*
LST_Max	−0.332	0.358	−0.925	0.355	−0.830	0.069	−11.99	< 0.001*
LST	−4.050	2.177	−1.861	0.062	−6.092	0.478	−12.74	< 0.001*
RAH	0.055	0.063	0.866	0.386	0.116	0.010	11.48	< 0.001*
Pp	0.008	0.093	0.092	0.926	0.086	0.011	7.822	< 0.001*
Infected *B. pfeifferi*								
*T* °C	0.408	1.237	0.33	0.74	−0.059	0.168	−0.349	0.727
pH	−1.33	104	−1.28	0.20	0.811	0.208	3.891	< 0.001*
TDS	0.054	0.256	0.21	0.83	0.142	0.047	3.077	0.002*
CE	0.032	0.134	0.24	0.81	0.065	0.023	0.827	0.005*
LST_Min	0.604	0.554	1.09	0.28	0.628	0.165	3.794	< 0.001*
LST_Max	−0.527	0.508	−1.04	0.301	−0.287	0.129	−2.214	0.029*
LST	−3.13	3.49	−0.90	0.37	3.304	0.705	4.686	< 0.001*
RAH	0.087	0.094	0.93	0.35	0.034	0.022	1.507	0.132
Pp	0.072	0.115	0.63	0.53	0.172	0.029	5.960	< 0.001*
Thermophilic coliforms								
*T* °C	−0.051	0.075	−0.69	0.490	−0.014	0.034	−0.423	0.672
pH	1.287	0.213	6.04	< 0.001*	−0.319	0.229	−1.395	0.163
TDS	−0.035	0.034	−1.03	0.3	0.015	0.014	1.074	0.283
EC	−0.020	0.017	−1.16	0.25	0.007	0.007	1.037	0.3
LST_Min	0.130	0.168	0.78	0.44	−0.023	0.083	−0.280	0.779
LST_Max	−0.073	0.167	−0.44	0.66	0.014	0.079	0.182	0.855
LST	0.959	1.128	0.85	0.40	0.925	0.511	1.811	0.070
RAH	0.012	0.028	0.42	0.67	−0.005	0.013	−0.414	0.679
Pp	0.014	0.030	0.5	0.62	0.001	0.013	0.087	0.931
*E. coli*								
*T* °C	−0.032	0.073	−0.44	0.66	0.014	0.034	0.423	0.672
pH	1.364	0.307	4.44	< 0.001*	0.319	0.229	1.395	0.163
TDS	−0.056	0.035	−1.56	0.117	−0.016	0.014	−1.074	0.283
EC	−0.030	0.018	−1.69	0.091	−0.007	0.007	−1.037	0.3
LST_Min	0.147	0.172	0.86	0.39	0.023	0.083	0.280	0.779
LST_Max	−0.084	0.170	−0.50	0.62	−0.014	0.079	−0.182	0.855
LST	0.030	1.143	0.03	0.98	−0.925	0.511	−1.811	0.070
RAH	0.016	0.028	0.57	0.57	0.006	0.013	0.41	0.679
Pp	0.0150	0.032	0.46	0.64	−0.001	0.014	−0.09	0.93

*T* °C: water temperature; pH: hydrogen potential; TDS: total dissolved solids; EC: electrical conductivity; LST_Min: minimum land surface temperature; LST_Max: maximum land surface temperature; LST: mean land surface temperature; RAH: relative air humidity; Pp: precipitation

*Indicates significant association at *P* < 0.05

### Human intestinal schistosomiasis

Epidemiological data were collected from 701 people aged 3–65 years in eight villages whose rivers were infested with *Biomphalaria* species. The POC-CCA test revealed *S. mansoni* antigens in the urine of 509 people (73.13%) (Table 4). In addition, *S. mansoni* eggs were observed in the feces of 81 of the 666 people examined (12.16%) using the KK test (Table 4). The prevalence varied significantly (*P* < 0.05) between 27% and 98.36% for the POC-CCA test and between 0 and 41.28% for the KK across all the villages studied (Table 4). Sahibly village had the highest rates of infected individuals (98.36% for POC-CCA and 41.28% for KK) and the highest CCA and KK means (454.73 ± 318.54 mV and 119.11 ± 272.97 EPG) (Table 4). Males (77.91% for POC-CCA and 13.90% for KK) and adults (96% for POC-CCA and 21% for KK) were significantly the most infected (*P* < 0.05); however, at the gender level, KK results were not significantly different. Adults had the highest mean CCA and EPG (289.88 ± 293.66 mV and 93.54 ± 262.66 EPG) (Table 4). Males had a higher average CCA (216.88 ± 260.78 mV), but females excreted more *S. mansoni* eggs (41.11 ± 196.23 EPG). However, the mean number of excreted eggs did not differ significantly between males and females (*P* > 0.05) (Table 4).

**Table 4:** Distribution of infection intensity based on POC-CCA and Kato-Katz in each group

Factors	Recruited	Tested POC-CCA	Pos POC-CCA	CCA	Tested KK	Pos KK	KK
Villages	*N*	*N*	*n* (%)	Mean ± SD (mV)	*N*	*n* (%)	Mean ± SD (EPG)
Domobly	99	99	77 (77.78)	119.04 ± 148.78	96	5 (5.21)	6.00 ± 28.07
Fouédougou	65	65	35 (53.85)	156.99 ± 260.39	65	9 (13.85)	38.40 ± 120.73
Francdougou	50	50	39 (78)	92.61 ± 65.10	50	0 (0.0)	0.00 ± 0
Golou	122	121	74 (61.16)	138.06 ± 217	122	9 (7.38)	36.20 ± 213.49
Pona	55	55	15 (27.27)	39.36.10 ± 83.60	40	0 (0.0)	0.00 ± 0
Sahibly	122	122	120 (98.36)	454.73 ± 318.54	109	45 (41.28)	119.11 ± 272.97
Yoya	126	126	93 (73.81)	154.40 ± 204.82	122	7 (5.74)	17.31 ± 86.02
Zéaglo	62	58	56 (96.55)	353.28 ± 258.10	62	6 (9.68)	24.77 ± 103.84
			*χ*^2^ = 137.43 *P* < 2.2e^−16^*	*χ*^2^ = 201.11 *P* = 2.2e^−16^*		*χ*^2^ = 137.43 *P* < 2.2e^−16^*	*χ*^2^ = 112.62 *P* = 2.2e^−16^*
Total	701	696	509 (73.13)	202.46 ± 259.58	666	81 (12.16)	36.03 ± 160.12
Sex							
M	345	344	268 (77.91)	216.68 ± 260.78	331	46 (13.90)	29.66 ± 111.07
F	346	342	236 (69.01)	192.90 ± 260.36	329	35 (10.64)	43.24 ± 198.92
			*χ*^2^ = 6.971 *P* = 0.0082*	*w* = 52,407 *P* = 0.01*		*χ*^2^ = 1.6277 *P* = 0.202	*w* = 52,746 *P* = 0.27
Age							
Adolescent [3–15]	591	588	405 (68.88)	188.16 ± 251.63	573	62 (10.82)	27.51 ± 136.83
Adult [16–65]	103	102	98 (96.08)	289.88 ± 293.66	89	19 (21.35)	93.54 ± 262.66
			*χ*^2^ = 32.552 *P* = 1.16e^−08^*	*w* = 20,355 *P* = 2.137e^−07^*		*χ*^2^ = 7.9513 *P* = 0.0048	*w* = 22,324 *P* = 0.0024*

* Indicates significant association at *P* < 0.05

### Spatial distribution of intestinal schistosomiasis risk

**Fig. 4﻿:**
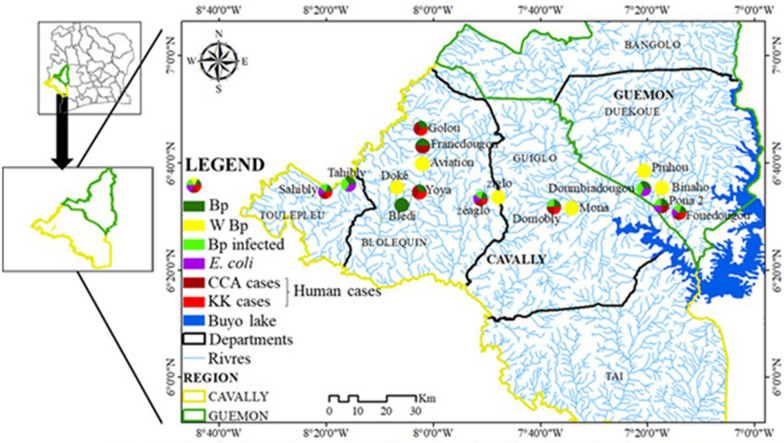
Distribution of malacological, microbiological, and human intestinal schistosomiasis data

Figure 4 shows the similarities in the distribution of *B. pfeifferi*, infected *B. pfeifferi*, *E. coli*, and human cases (with CCA and *S. mansoni* eggs in the stool). Human cases of schistosomiasis were detected in all locations where *B. pfeifferi* was reported. Although a human diagnosis of schistosomiasis was not made in Tahibly and Doumbiadougou, the risk of infection of the populations living along the rivers surveyed was confirmed by the presence of infected *B. pfeifferi*. A correlation test between human infections and the abundance of *Biomphalaria* showed a strong positive correlation between these two variables (*r*^2^ = 0.879, *t* = 3.2, *P* = 0.04959).

## Discussion

Each bilharzia endemic area has biological, ecological, social, and economic characteristics that may differ from one site to another [4]. These characteristics may therefore affect the disease spread process differently.

In order to gain a deeper understanding of the persistence of intestinal bilharzia in the western zone of Côte d’Ivoire, an environmental survey was conducted to characterize and evaluate the influence of physicochemical parameters of water and climatic factors on the presence and proliferation of the intermediate host (*B. pfeifferi*) and its infection by *S. mansoni*.

The malacological survey revealed the presence of *B. globosus* and *B. pfeifferi* and confirmed the recent presence of *B. forskalii*, which are intermediate hosts of bilharzia. These results corroborate those of previous studies [7, 33] which observed the same snail species. However, the presence of *B. forskalii* is very recent. This study is the second to note the presence of this species in this part of Côte d'Ivoire. *Bulinus globosus* and *B. forskalii* had not been found to excrete schistosomes [33]. This study also highlights the sanitary danger posed by the presence of *B. pfeifferi* infected with the *S. mansoni* parasite of intestinal bilharzia. The prevalence observed varied, and was as high as 100% in certain rivers and localities, notably those of Toulepleu. This prevalence could be the result of environmental conditions favorable to the survival and infectious capacity of the parasite. Studies have reported the impact of environmental factors such as pH and water temperature on the infection of snails [34–36]. Indeed, the combination of these two factors has been suggested to impact the physiology and maturation rates of *S. mansoni* within *Biomphalaria* [37, 38]. Microbiological assessment of water quality represents a critical tool for preventing water-borne diseases. In the case of bilharziasis, it allows us to highlight contamination of human fecal origin in the habitats of *Bulinus* and *Planorbis*. It is in this habitat contaminated by coliforms (aquatic environment) that the contamination of snails and humans essentially occurs [4]. Analyses of the water samples revealed contamination with total coliforms and thermophilic coliforms (*E. coli*). These coliforms would have reached the ponds through the dumping or drainage of human waste, as these bacteria are adapted to the human intestinal tract. This can be explained by the presence of human waste around some of the ponds surveyed, which therefore indicates a lack of sanitation and toilets. Thus, the observed infection of the snails implies that they would have been in contact with human waste containing *S. mansoni* eggs.

The temperature range (21–32 °C) recorded during this study is characteristic of the water temperature in tropical zones [7, 39]. The waters in the study area were acidic (pH 1 to 6.9), which could be due to the use of nitrogen fertilizers [40], CO_2_ from the decomposition of aquatic organic matter [41], and the type of rocks present, especially acidic rocks [42]. The EC and TDS values remained within the range of values for fresh water. They are also similar to those reported by previous studies conducted in the same area [33, 39]. These values are thought to be due to low sodium and chloride ion levels and the size of the catchment [43]. The values recorded for climatic factors remained within the range of values recorded in previous studies [33], which are characteristic of tropical zones.

Regression between physicochemical parameters and the abundance of *B. pfeifferi* revealed no significant associations. However, significant associations with the presence of snails were detected—in particular, a positive association was found with water temperature. Indeed, high water temperatures favor the increase in aquatic plants, which allows the development and thus the availability of food, as noted in previous studies [44, 45]. A negative association was observed between pH and the presence of *B. pfeifferi*. This could be explained by the fact that the pH range found in this study would slow the defense system [46], reproduction, and maturation of *Biomphalaria* [7]. Manyangadze et al. and Rowel et al. [13, 38] found similar results supporting a negative association between pH and *Biomphalaria* along Lake Albert and Lake Victoria.

TDS and EC demonstrated negative associations with the presence and abundance of snails. Indeed, the presence and number of *Biomphalaria* decreased with the TDS and EC values of the surveyed water bodies. Other studies have also reported negative associations between *Biomphalaria*, EC, and TDS [38, 47]. Climatic factors LST and LST_Max) were negatively associated, and Pp, RAH, and LST_Min were positively associated with the presence of *Biomphalaria*. These results indicate that some of these factors are inhibitors and others are activators of mollusk reproduction [13, 48]. A close relationship exists between climatic factors and water temperature [28]. The high temperatures at the surface of the earth cause a decrease in the surface water temperatures, which leads to a decrease in the snail population [49]. The pH, TDS, EC, LST_Min, LST, high RAH, and high Pp would favor shellfish infection. Indeed, pH and water temperature affect the physiology and maturation rates of the parasite within the vector but also influence the development and survival of the cercarial stages [37]. Similar results were reported by Rowel et al. [38].

Furthermore, *Biomphalaria* infection was influenced by the abundance of thermophilic coliforms in the surveyed water bodies. The human infection rate recorded in this study indicates the hyperendemicity of *S. mansoni* infection. This rate is further representative of the level of risk represented by the environmental factors examined in addition to the positive correlation between shellfish and human infection in this study. Thus, the locations with the highest rates of infection and the highest infection intensity were characterized by the simultaneous presence of *B. pfeifferi* and fecal contamination. The risk of infection is therefore very high in the villages of Sahibly, Zéaglo, and Fouédougou, whereas this risk is not as high in the other localities.

## Conclusions

This study revealed that almost all the physicochemical and climatic factors considered in this study (apart from the *T* °C and RAH of the area for infected *Biomphalaria*) influenced the *Biomphalaria* population and its infection. Thermophilic coliforms, meanwhile, were influenced only by pH. This reflects the environmental conditions that are conducive to the maintenance of infection in this part of the country. The high rates of human infection are a direct consequence of this. In order to control schistosomiasis most effectively, greater efforts are needed to clean up the environment, treat people living in the area according to the risk level of the locality, and raise awareness of the disease. The results of this study show that despite control efforts, intestinal schistosomiasis remains endemic or hyperendemic, depending on the locality in the western zone studied. This calls for urgent and assiduous control measures that are more specific and based on the level of endemicity of the disease, taking into account the entire population and not just a subset of it. In addition, the presence of *B. forskalii*, previously absent from the area, represents a risk of spreading urinary bilharziasis, which was previously endemic to areas outside the western part of the country. This could make the control effort more difficult.

## Data Availability

All relevant data are contained within the manuscript.
